# Identification and genetic analysis of candidate genes for resistance against *Phytophthora sojae* in soybean using a genome-wide association study

**DOI:** 10.3389/fpls.2025.1520999

**Published:** 2025-05-02

**Authors:** Hye Rang Park, Su Vin Heo, Beom Kyu Kang, Hyoseob Seo, Eunsoo Lee, Jihee Park, Yun Woo Jang, Jeong Hyun Seo, Girim Park, Jun Hoi Kim, Yeong Hoon Lee, Won Young Han, Myung Chul Seo, Ji-Ung Jeung

**Affiliations:** ^1^ Division of Upland Crop Breeding Research, Department of Southern Area Crop Science, National Institute of Crop Science, Rural Development Administration, Miryang, Republic of Korea; ^2^ Division of Crop Production Technology Research, Department of Southern Area Crop Science, National Institute of Crop Science, Rural Development Administration, Miryang, Republic of Korea

**Keywords:** soybean, *Phytophthora sojae*, genome-wide association study, Phytophthora root and stem rot, nucleotide-binding site leucine-rich repeat, serine-threonine protein kinases

## Abstract

Phytophthora root and stem rot (PRSR), an infection caused by *Phytophthora sojae* in soybean [*Glycine max* (L.) Merr.], is an important threat to soybean production in South Korea; however, the precise genetic mechanisms related to PRSR resistance remain largely unclear, limiting the development of resistant cultivars. This study aimed to identify candidate resistance (*R*) genes related to PRSR resistance in soybeans through a genome-wide association study (GWAS). We evaluated 205 soybean accessions inoculated with *P. sojae* isolates GJ3053 or AD3617 using the hypocotyl inoculation method and genotyped them using a 180K Axiom SoyaSNP chip. We identified 19 significant single-nucleotide polymorphisms (SNPs) related to resistance against one or both of the two isolates: GJ3053 resistance was associated with SNPs on chromosomes 2, 3, 9, 10, 14, and 16, whereas AD3617 resistance was related to SNPs on chromosomes 3, 13, and 16. The SNP AX-90410433 (3,628,549 bp) on chromosome 3 was significantly linked to resistance against both isolates, based on the linkage disequilibrium (480 kb) and –log_10_(*p*) values (6.62). This region harbors key resistance gene analogs (RGAs), including nucleotide-binding site leucine-rich repeat and serine-threonine protein kinases. Among the 34 identified RGAs in the 2.9**–**4.4 Mbp region, G*lyma.03g036500*, which encodes a protein with serine kinase activity, emerged as a strong candidate. Haplotype analysis revealed that this gene exhibited genotypic patterns consistent with the resistance phenotypes of the selected accessions. Validation through gene expression and kompetitive allele-specific PCR marker analysis supported the role of G*lyma.03g036500* in PRSR resistance. These findings underscore the significance of identifying and utilizing PRSR resistance genes, such as *Glyma.03g036500*, to enhance pathogen resistance in soybean breeding programs. Our results can inform the development of cultivars with improved resistance to *P. sojae*, thus potentially mitigating the effect of pathogenic stress on crop productivity and quality, and contributing to sustainable agriculture.

## 1 Introduction

Soybean [*Glycine max* (L.) Merr.] is a globally important crop with high protein and oil contents. Several traditional soy-based foods, such as soy milk, soy sauce, and tofu, are integral to Asian diets. The recent increase in the global consumption of soy products has greatly impacted the protein market (Henchion et al., 2017; Gupta and Manjaya, 2022; Qin et al., 2022; Liu et al., 2023). However, global yield is greatly limited by several soybean diseases, such as Asian soybean rust (*Phakopsora pachyrhizi*), sudden death syndrome (*Fusarium virguliforme*), bacterial blight (*Pseudomonas syringae* pv. *glycinea*), and Phytophthora root and stem rot (PRSR; *Phytophthora sojae*) (Hosseini et al., 2023), among which PRSR is one of the most severe diseases.

PRSR, caused by the soil-borne oomycete *P. sojae* Kaufmann and Gerdemann, was initially documented in Indiana, USA (Kaufmann and Gerdemann, 1958). *P. sojae* is a self-fertile pathogen and predominantly infects soybeans. Under high moisture conditions, oospores germinate into mycelia, which produce sporangia and zoospores (Dorrance et al., 2007). Zoospores are chemotactically attracted to soybean roots through root exudates such as daidzein and genistein. Then, they invade the roots, germinate, and spread into the root tissue (Schmitthenner, 1985; Morris and Ward, 1992; Dorrance, 2018). This can cause seed decay and seedling damping-off during the early stages of soybean growth. In highly susceptible cultivars, brown lesions may appear on stems at later growth stages, and plant tissues collapse, thus impairing plant health and yield (Schmitthenner, 1985).

Soybean may exhibit resistance (*R*) gene-mediated and quantitative resistance against *P. sojae* infection (Schmitthenner, 1985). *R* gene-mediated resistance is primarily achieved through single dominant genes, known as resistance to *P. sojae* (*Rps*) genes, whereas quantitative resistance involves multiple genes, including quantitative trait loci (QTLs), that provide partial and durable resistance against a wide range of *P. sojae* races (Jang and Lee, 2020). The *Rps* gene in soybeans plays a crucial role in regulating PRSR by mediating a race-specific defense response through a gene-for-gene relationship with the avirulence (*Avr*) gene in *P. sojae* (Flor, 1971; Dorrance, 2018; Dodds, 2023). More than 30 *Rps* alleles/genes have been reported on 11 soybean chromosomes, of which approximately 70% are located on chromosomes 3, 13, and 18 (Jang and Lee, 2020; Chandra et al., 2022). Specifically, *Rps1a, 1b, 1c, 1d*, and *1k* and approximately 20 *Rps* alleles (*Rps7, 9, UN1, Yu25, YD25, YD29, HN, Q, WY, HC18, X, GZ, unnamed in* cv.*Waseshiroge, DW*, *SDB*, *T1, T2, T3*, and *14*) have been mapped to chromosome 3 (Demirbas et al., 2001; Weng et al., 2001; Sugimoto et al., 2008, 2011; Sun et al., 2011; Wu et al., 2011; Lin et al., 2013; Zhang et al., 2013; Cheng et al., 2017; Li et al., 2017; Niu et al., 2017; Zhong et al., 2018, 2019; Jang et al., 2020a; Jiang et al., 2020; Chen et al., 2021; Matsuoka et al., 2021; You et al., 2023b; Bolaños-Carriel et al., 2022; Clevinger et al., 2024; Hodge et al., 2024). Other *Rps* alleles have been identified on chromosomes 13 (*Rps3a*, *3b, 3c*, *SN10*, and *CD*), 18 (*Rps4, 5, 6*, *JS*, *12*, and *13*) (Demirbas et al., 2001; Gordon et al., 2007; Sugimoto et al., 2012; Sahoo et al., 2017), 2, 7, 10, 16, 17, 19, and 20 (Jang and Lee, 2020; Chandra et al., 2022). These alleles, found in gene-dense regions, are located near genes encoding nucleotide binding site-leucine-rich repeat (NBS-LRR) proteins and serine/threonine protein kinases (STKs), which are considered resistance gene analogs (RGAs) (Sekhwal et al., 2015; Niu et al., 2017; Sahoo et al., 2017; Zhong et al., 2018; Jang et al., 2020a; Jiang et al., 2020; Karhoff et al., 2022; You et al., 2023b).

PRSR was first reported in Chungnam Province, South Korea, in 1996 (Jee et al., 1998). In 2019, the effects of four *P. sojae* isolates were assessed on 20 major Korean soybean cultivars (Kang et al., 2019). Genetic regions associated with resistance were identified on chromosome 3 (3.8**–**4.6 Mbp) in the Daepung/Daewon RIL population (Jang et al., 2020a). Additionally, resistance was related to the 3.3**–**4.3 Mbp regions on chromosome 3 in the Daepung/Saedanbaek RIL population (You et al., 2023b). These regions overlapped by 402 kbp and contained six NBS-LRR genes. The 36.2–37.4 Mbp region on chromosome 3 was also associated with resistance in the Daepung/Socheong2 RIL population (Jang et al., 2020b). On chromosome 18, a resistance region was identified at 2.1–2.6 Mbp in the Daepung/Socheong2 RIL population (Jang et al., 2020b) and at 55.9–56.4 Mbp in the Daepung/Cheonal RIL population (You et al., 2023a).

New developments such as genome-wide association studies (GWAS) have greatly advanced research into the genetic loci linked to disease resistance in soybean. By incorporating high-density single nucleotide polymorphism (SNP) markers and advanced statistical models, GWAS allows for high-resolution mapping of resistance genes across diverse populations. GWAS has been used to map both *Rps*-genes and quantitative resistance to *P. sojae* (Schneider et al., 2016; Qin et al., 2017; Luke et al., 2019; Rolling et al., 2020; Van et al., 2021; Chandra et al., 2022; You et al., 2024a, 2024b). This approach enables breeders to develop molecular markers for more efficient selection of resistant resources.

The present study investigated the specific genetic loci and candidate genes linked with PRSR resistance in soybeans using GWAS. Accordingly, we genotyped 205 diverse soybean accessions using a 180K Axiom SoyaSNP chip and evaluated their resistance to two *P. sojae* isolates, GJ3053 and AD3617. Candidate *R* genes were discovered within significant genomic areas and validated using gene expression and kompetitive allele-specific PCR (KASP) markers analyses. This study advances the current paradigm of the genetic mechanisms underlying PRSR resistance and provides valuable resources for designing breeding strategies that boost resistance to pathogen-induced stress.

## 2 Materials and methods

### 2.1 Plant and pathogen materials

The 205 soybean accessions included 170 Korean cultivars, 15 breeding lines, and 20 landraces (**Supplementary Table 1**). Seeds were harvested at the Southern Crop Department of the National Institute of Crop Science in Miryang, South Korea (35° 29’ 46.5” N, 128° 44’ 29.9” E) in 2019. The *P. sojae* isolates were obtained from Andong (AD3617) and Gimje (GJ3053), South Korea (Korean Agricultural Culture Collection; 48988 and 48989, respectively) (Heo et al., 2024).

### 2.2 Phenotypic assay of resistance to *P. sojae*

The pathotypes of the two isolates were determined using 16 soybean differentials: GJ3053 (*vir1a, 1b, 1c, 1d, 1k, 2, 3a, 3b, 3c, 4, 5, 6, 7*, and *8*) and AD3617 (*vir1a, 1b, 1d, 3a, 3b, 3c, 4, 5, 6, 7*, and *8*) (Heo et al., 2024). In 2021, the resistance of 205 accessions to GJ3053 and AD3617 was evaluated using the hypocotyl inoculation method (Dorrance et al., 2004; Heo et al., 2024). To prepare inoculum, mycelial fragments of *P. sojae* were cultured on a 10% V8 agar medium at 28°C for 7 days. Twelve seedlings per accession were grown in 13 cm plastic pots placed in a greenhouse for 7 days. A 1 cm incision was made on the hypocotyl below the cotyledons using a scalpel. The wound site was inoculated with 0.2–0.4 mL of slurry which was injected using a 10-mL syringe with an 18-gauge needle. The inoculated seedlings were maintained under humid conditions for 24 hours. Resistance was evaluated based on the survival rates of seedlings 7 days post-inoculation. The survival rate was calculated as the proportion of surviving seedlings relative to the total number inoculated. Accessions were classified as susceptible (S) when the survival rate was below 20%, intermediate (I) when it was between 20% and 80%, and resistant (R) when it was above 80%. The experiment was conducted in three independent replicates.

### 2.3 DNA extraction and genotyping

Genomic DNA was extracted from the leaves using a Maxwell RSC 48 automatic nucleic acid extraction device (Promega, Madison, WI, USA). The quality of the extracted DNA was evaluated using a NanoDrop ND-2000 spectrophotometer (Thermo Fisher Scientific, Waltham, MA, USA). Genotyping of the 205 accessions was performed using a 180K Axiom SoyaSNP chip (Affymetrix, Santa Clara, CA, USA) (Lee et al., 2015), a high-throughput genotyping platform. Among the 205 accessions, ten accessions were subjected to genome resequencing using a HiSeq X platform (Illumina, San Diego, CA, USA): Cheongja4, Cheongja5, Daepung2, Seonpung, Seonyu2, Taekwang, Cheongja2, Jungmo3009, Heugmi, and Namcheon.

### 2.4 GWAS

GWAS was performed using Trait Analysis by association, Evolution, and Linkage (TASSEL) software v5.2.89 with a generalized linear model [GLM, principal component analysis (PCA)] and mixed linear model (MLM; PCA + K). The SNPs in the scaffold regions with a minor allele frequency (MAF) < 5 or 10% were removed to ensure genotype quality. Finally, of the total 180,375 SNPs, 79,102 (MAF < 5%) and 65,128 (MAF < 10%) determined by GLM and MLM were used to estimate linkage disequilibrium (LD) (**Supplementary Table 2**). The threshold for significant association was set to *p*=1/n (Seo et al., 2023), where n is the number of markers, resulting in a –log_10_(*p*) > 5. A quantile-quantile (QQ) plot was analyzed using TASSEL v5.2.89, and a Manhattan plot was produced using R v4.3.2. Allele frequency correlation (*r^2^*) analysis was performed using the TASSEL (Bradbury et al., 2007). The point of LD decay was defined as the interval where *r^2^* decreased to half its peak (Lam et al., 2010).

### 2.5 Identification of candidate genes annotation and haplotype analysis

SoyBase (www.soybase.org, accessed May 2024) was used to investigate the significant SNPs through GWAS and identify candidate genes. Using the soybean reference genome (Wm82.a2.v1), candidate genes were identified based on RGAs within the LD region of the significant SNPs identified by GWAS. For haplotype analysis, we selected accessions with the most significant differences in survival rates for GJ3053 and AD3617: [Cheongja2 (R), Heugmi (R), Jungmo3009 (R), and Namcheon (R)] and [Cheongja4 (S), Cheongja5 (S), Daepung2 (S), Seonpung (S), Seonyu2 (S), and Taekwang (S)], respectively (**Supplementary Table 3**). Sequence alignment was conducted to compare resistant with susceptible groups, thereby identifying SNP variants specific to each group within RGAs. Based on these variants, haplotypes were classified and analyzed for their association with resistance.

### 2.6 Expression analyses of candidate genes

Seven days after planting, Jungmo3009 (R) and Seonpung (S) were treated with isolates GJ3053 and AD3617 using the hypocotyl inoculation method (Dorrance et al., 2004; Heo et al., 2024). Samples (1cm) from both above and below the treated hypocotyl were collected at 0, 6, 12, 24, 48, and 72 hours after inoculation, and stored at -80°C. Total RNA was extracted using an RNeasy PowerPlant Kit (Qiagen, Hilden, Germany), and cDNA was generated using RNA-to-cDNA EcoDry (Takara, Shiga, Japan). Primers for the quantitative real-time polymerase chain reaction (qRT-PCR) were designed based on the conserved coding region using Primer3 (**Supplementary Table 4**). Gene expression was determined by qRT-PCR using SsoAdvanced Universal SYBR Green Supermix (Bio-Rad Laboratories, Hercules, CA, USA) on a QuantStudio5 (Thermo Fisher Scientific, MA, USA). The PCR conditions included an initial step at 50°C for 2 min, followed by denaturation at 95°C for 10 min, and 40 cycles at 95°C for 15 sec, 63°C for 1 min, and 72°C for 15 sec. The housekeeping gene *Actin 11* was used as an internal control. We confirmed the infection of the inoculated soybean samples based on the expression of pathogenesis-related (*PR*) genes (*GmPRP* and *GmERF113*) (Jiang et al., 2015; Zhao et al., 2017). The 2^-ΔΔCT^ method was applied to estimate the relative expression levels of candidate genes (Livak and Schmittgen, 2001). Three biological replicates, each with three technical replicates, were performed for each sample. Student’s *t*-test was conducted to assess the differences in gene expression between Jungmo3009 (R) and Seonpung (S).

### 2.7 Kompetitive allele specific PCR (KASP) markers validation

The target region in the candidate gene was developed for KASP markers (**Supplementary Table 5**). The KASP markers were designed by LGC genomics (London, UK) using 100 bp of flanking sequences, with two allele-specific forward primers and one shared reverse primer. Genotyping was performed using the QuantStudio 5 (Thermo Fisher, MA, USA). The amplification conditions included an initial step at 94°C for 15 min, followed by 10 cycles of 94°C for 20 sec and 61°C for 1 min, then 26 cycles of 94°C for 20 sec and 55°C for 1 min.

## 3 Results

### 3.1 Phenotype analysis of *P. sojae* isolates GJ3053 and AD3617

**Figure 1:**
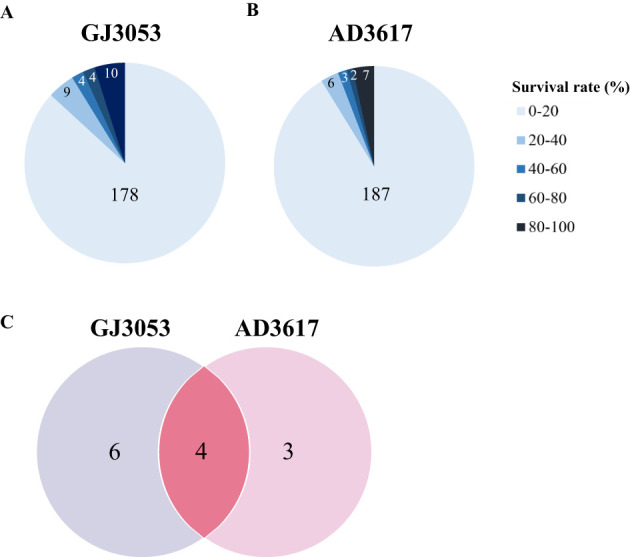
The distribution of survival rates of 205 soybean accessions after infection with *Phytophthora sojae* isolates GJ3053 **(A)** and AD3617 **(B)** was analyzed. The number of accessions within each survival rate range is indicated by the corresponding values shown inside the chart sections. **(C)** The distribution of resistant accessions (survival rate ≥ 80%) to each isolate was also examined. The number represent the count of accessions exhibiting resistance to either isolate individually or to both isolates.

Following inoculation with isolates GJ3053 and AD3617, 178 and 187 accessions, respectively, showed survival rates below 20% and were classified as susceptible (**Figures 1A, B**, **Supplementary Table 6**). Survival rates of 20–40% were observed in nine (GJ3053) and six (AD3617) accessions, 40–60% in four and three accessions, and 60–80% in four and two accessions, respectively. Accessions with a survival rate above 80% were classified as resistant. Ten accessions were resistant to GJ3053 (Blackhawk, Gwanggyo, Namcheon, Saeal, Cheongja2, Heugmi, Socheong2, Jungmo3009, Sobeaknamul, and Taecheong), and seven were resistant to AD3617 (Namcheon, Manpoong, Cheongja2, Heugmi, Heugsung, Jungmo3009 and Miso). Among them, Cheongja2, Heugmi, Jungmo3009, and Namcheon showed resistance to both GJ3053 and AD3617 (**Figure 1C**; **Supplementary Table 3**).

### 3.2 GWAS for soybean resistance to *P. sojae* isolates

**Figure 2:**
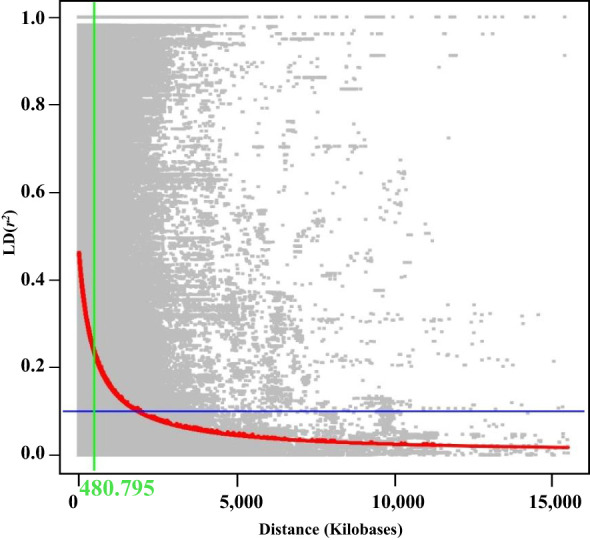
Linkage disequilibrium (LD) decay analysis of 205 soybean accessions based on pairwise *r^2^* values. Green line: position where LD decay reaches half of its maximum value, red line: rate of LD decay, blue line: standard LD threshold (*r^2^* = 0.1).

**Figure 3:**
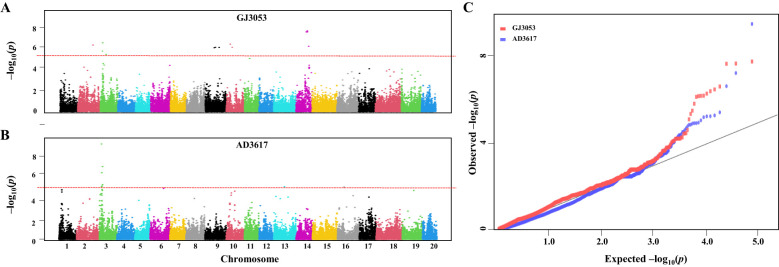
Genome-wide association study (GWAS) of *P. sojae* resistance genes in 205 soybean accessions. Manhattan plot of GWAS for *P. sojae* GJ3053 **(A)** and AD3617 **(B)**. Red horizontal dashed line: significance threshold: -log_10_(*p*)= 5. **(C)** Quantile-quantile (QQ) plots of GWAS for the two isolates.

We conducted a GWAS based on identified SNPs to estimate the genomic diversity among the accessions. The initial *r*² value decreased by half at 480,795 bp (**Figure 2**). To identify SNPs associated with resistance to GJ3053 and AD3617, GLM and MLM were employed (**Supplementary Figures 1**, **2**). MLM methods with MAF < 10% were selected based on our comparison of the Manhattan and QQ plots (**Supplementary Table 2**, **7**). Significant associations were identified at *p*-value threshold 1/n, where n represents the total number of SNPs, resulting in a –log_10_(*p*) > 5 (**Supplementary Table 2**). Using this threshold, we identified 14 and six loci were associated with isolates GJ3053 and AD3617, respectively (**Figure 3**; **Table 1**). The significant SNPs were located on chromosomes 2, 3, 9, 10, 14, and 16 for GJ3053 and 3, 13, and 16 for AD3617. In GJ3053, the –log_10_(*p*) of AX-90438121 was 6.36 on chromosome 2 and those for AX-90354028, AX-90410433, and AX-90339964 were 5.79, 6.58, and 5.48 on chromosome 3, respectively. The –log_10_(*p*) values for AX-90395336, AX-90347843, and AX-90482872 were 6.27, 6.33, and 6.14, respectively, on chromosome 9, and those for AX-90467453 and AX-90331570 were 6.64 and 6.31, respectively, on chromosome 10. The –log_10_(*p*) of AX-90327146, AX-90397007, AX-90391625, and AX-90399355 were 7.72, 7.61, 7.62, and 6.26, respectively, on chromosome 14. In AD3617, the –log_10_(*p*) of AX-90432113, AX-90410433, AX-90402933, and AX-90365087 were 9.43, 6.59, 5.40, and 7.19, respectively, on chromosome 3. The –log_10_(*p*) value for AX-90525316 was 5.22 on chromosome 13, and that for AX-90449650 was 5.18 on chromosome 16 (**Table 1** and **Figure 3**). The most significant SNP for GJ3053 and AD3617 was AX-90410433 on chromosome 3. Based on the LD decay distance, the LD was estimated at 480 kb upstream to downstream of each SNP (**Figure 2**). The LD for AX-90410433 (3,628,549 bp, W82.a2.v1) occurred at 3,147,754**–**4,109,344 bp.

**Table 1:** Significant SNPs associated with *P. sojae* GJ3053 and AD3617 in soybean identified by GWAS with MLM.

Isolate	Chr^1^	SNP	Physical positions (bp) ^2^	Linkage disequilibrium block (bp)	–log_10_(*p*)	Marker *R^2^*	No. of ORFs^3^
GJ 3053	2	AX-90438121	34,224,450	33,743,655-34,705,245	6.36	0.156	12
	**3**	AX-90354028	3,417,978	2,937,183-3,898,773	5.79	0.142	69
		**AX-90410433**	**3,628,549**	**3,147,754-4,109,344**	6.58	0.160	62
		AX-90339964	15,215,000	14,734,205-15,695,795	5.48	0.136	2
	9	AX**-**90395336	20,563,970	20,083,175-21,044,765	6.27	0.148	15
		AX**-**90347843	31,690,000	31,209,205-32,170,795	6.33	0.152	17
		AX**-**90482872	40,718,457	40,237,662-41,199,252	6.14	0.151	96
	10	AX**-**90467453	9,112,938	8,632,143-9,593,733	6.64	0.158	35
		AX-90331570	13,685,110	13,204,315-14,165,905	6.31	0.150	18
	14	AX-90327146	18,058,454	17,577,659-18,539,249	7.72	0.190	14
		AX-90397007	19,400,109	18,919,314-19,880,904	7.61	0.188	16
		AX-90391625	35,814,319	35,333,524-36,295,114	7.62	0.188	12
		AX-90399355	40,454,708	39,973,913-40,935,503	6.26	0.152	15
	16	AX-90331170	3,163,817	2,683,022-3,644,612	5.35	0.139	100
AD 3617	**3**	AX-90432113	3,285,331	2,804,536-3,766,126	9.43	0.212	67
		**AX-90410433**	**3,628,549**	**3,147,754-4,109,344**	6.59	0.161	62
		AX-90402933	5,210,627	4,729,832-5,691,422	5.40	0.110	69
		AX-90365087	5,287,030	4,806,235-5,767,825	7.19	0.155	67
	13	AX-90525316	30,033,688	29,552,893-30,514,483	5.22	0.125	131
	16	AX-90449650	8,750,924	8,270,129-9,231,719	5.18	0.124	33

^1^Chr, Chromosome. ^2^Physical positions are based on soybean genome W82.a2.v1.(http://soybase). ^3^ORFs: open reading frames. The bolded SNPs indicate an overlapping region on chromosome 3 for both the GJ3053 and AD3617 isolates.

### 3.3 Identification of candidate resistance genes

Genes with homology to canonical plant disease resistance genes, such as those encoding LRR and STKs family proteins, were identified in significant genomic regions. In GJ3053, one gene with homology to known resistance genes was detected on chromosome 2, seven on chromosome 3, ten on chromosome 9, four on chromosome 10, five on chromosome 14, and two on chromosome 16 (**Table 2**). In AD3617, 14 genes were identified on chromosome 3, six on chromosome 13, and five on chromosome 16. Based on the GWAS results for chromosome 3, we expanded the region around AX-90410433 (3,628,549 bp), which overlapped in both GJ3053 and AD3617, to 3.1–4.1 Mbp to identify RGAs. As a result, we identified candidate genes related to resistance to *P. sojae*: *Glyma.03g027200*, *Glyma.03g032300*, *Glyma.03g033900*, *Glyma.03g034200, Glyma.03g036000*, and *Glyma.03g036500*. Through this analysis, we determined that the region containing candidate genes on chromosome 3 spanned the 2.9–4.4 Mbp region (**Table 2**), which overlaps with the previously reported *Rps1*, *RpsDW*, and *RpsSDB* regions (Demirbas et al., 2001; Jang et al., 2020a; You et al., 2023b).

**Table 2:** Candidate genes associated with resistance to *P. sojae* GJ3053 and AD3617.

Isolate	Chr^1^	Gene	Physical position^2^ (bp)		Annotation
			Start	End	
GJ 3053	2	*Glyma.02g186200*	33,549,176	33,550,404	Protein serine/threonine kinase activity
	3	*Glyma.03g027200*	2,996,616	2,999,968	Leucine-rich repeat receptor-like protein kinase family protein
		*Glyma.03g032300*	3,690,272	3,692,978	Leucine-rich repeat receptor-like protein kinase
		*Glyma.03g033900*	3,952,740	3,954,416	Regulation of cyclin-dependent protein serine/threonine kinase
		*Glyma.03g034200*	3,989,072	3,993,561	Leucine-rich repeat protein kinase family protein
		*Glyma.03g036000*	4,335,981	4,341,371	Protein serine/threonine/tyrosine kinase activity
		*Glyma.03g036500*	4,402,746	4,408,323	Protein serine/threonine kinase activity
		*Glyma.03g069800*	15,703,649	15,719,178	SERINE/THREONINE-PROTEIN KINASE UNC-51-RELATED
	9	*Glyma.09g109100*	21,233,142	21,236,812	Transmembrane receptor protein serine/threonine kinase activity
		*Glyma.09g110500*	21,870,064	21,872,625	Protein serine/threonine kinase activity
		*Glyma.09g110700*	21,937,240	21,939,255	Protein serine/threonine kinase activity
		*Glyma.09g177600*	40,214,417	40,227,685	Protein serine/threonine kinase activity
		*Glyma.09g178500*	40,336,748	40,341,928	Cyclin-dependent protein serine/threonine kinase activity
		*Glyma.09g179200*	40,391,988	40,396,974	Protein serine/threonine phosphatase activity
		*Glyma.09g181400*	40,361,573	40,635,776	Protein serine/threonine kinase activity
		*Glyma.09g181500*	40,640,404	40,642,456	Protein serine/threonine kinase activity
		*Glyma.09g181600*	40,649,262	40,660,028	Protein serine/threonine kinase activity
		*Glyma.09g185700*	41,075,512	41,084,216	Protein serine/threonine phosphatase activity
	10	*Glyma.10g096600*	15,335,173	15,341,113	Calcium-dependent protein serine/threonine kinase activity
		*Glyma.10g096800*	15,343,764	15,361,742	Calcium-dependent protein serine/threonine kinase activity
		*Glyma.10g098400*	17,004,342	17,007,423	Protein serine/threonine kinase activity
		*Glyma.10g100600*	18,396,083	18,399,163	Receptor-like serine/threonine-protein kinase
	14	*Glyma.14g124400*	19,061,201	19,065,410	Leucine-rich repeat protein kinase family protein
		*Glyma.14g124700*	19,277,952	19,281,950	Leucine-rich repeat transmembrane protein kinase family protein
		*Glyma.14g164400*	40,515,203	40,522,203	Protein serine/threonine kinase activity
		*Glyma.14g165700*	40,916,648	40,932,983	Protein serine/threonine kinase activity
		*Glyma.14g166000*	40,950,477	40,961,355	Protein serine/threonine kinase activity
	16	*Glyma.16g032700*	3,094,789	3,099,121	Protein serine/threonine kinase activity
		*Glyma.16g034900*	3,273,872	3,276,746	Receptor-like serine/threonine-protein kinase
AD 3617	3	*Glyma.03g026800*	2,935,639	2,938,424	Leucine-rich repeat receptor-like protein kinase family protein
		*Glyma.03g027200*	2,996,616	2,999,968	Leucine-rich repeat receptor-like protein kinase family protein
		*Glyma.03g032300*	3,690,272	3,692,978	Leucine-rich repeat receptor-like protein kinase
		*Glyma.03g033900*	3,952,740	3,954,416	Regulation of cyclin-dependent protein serine/threonine kinase
		*Glyma.03g034200*	3,989,072	3,993,561	Leucine-rich repeat protein kinase family protein
		*Glyma.03g036000*	4,335,981	4,341,371	Protein serine/threonine/tyrosine kinase activity
		*Glyma.03g036500*	4,402,746	4,408,323	Protein serine/threonine kinase activity
		*Glyma.03g036900*	4,497,651	4,498,973	Protein serine/threonine kinase activity
		*Glyma.03g037200*	4,558,538	4,560,610	Serine/Threonine protein kinase 10
		*Glyma.03g038900*	4,807,180	4,809,600	Protein serine/threonine kinase activity
		*Glyma.03g043100*	5,448,239	5,450,293	Protein serine/threonine phosphatase activity
		*Glyma.03g043700*	5,523,404	5,525,620	Protein serine/threonine phosphatase activity
		*Glyma.03g043900*	5,534,982	5,539,691	LRR and NB-ARC domains-containing disease resistance protein
		*Glyma.03g044100*	5,575,689	5,577,750	Protein serine/threonine kinase activity
	13	*Glyma.13g181400*	29,466,548	29,471,275	Protein serine/threonine kinase activity
		*Glyma.13g184200*	29,789,364	29,800,000	Serine/threonine kinase activity
		*Glyma.13g187600*	30,134,637	30,143,817	Serine/threonine kinase activity
		*Glyma.13g188800*	30,236,854	30,239,730	Protein serine/threonine kinase activity
		*Glyma.13g188900*	30,244,450	30,249,360	Protein serine/threonine kinase activity
		*Glyma.13g189000*	30,251,712	30,254,980	Protein serine/threonine kinase activity
	16	*Glyma.16g078800*	8,103,196	8,107,130	Protein serine/threonine kinase activity
		*Glyma.16g078900*	8,118,602	8,122,477	Protein serine/threonine kinase activity
		*Glyma.16g079000*	8,126,768	8,129,107	Protein serine/threonine kinase activity
		*Glyma.16g079200*	8,183,660	8,187,220	Receptor serine/threonine kinase binding
		*Glyma.16g081700*	8,795,648	8,797,703	Serine/threonine kinase activity

^1^Chromosome. ^2^Physical positions are based on soybean genome W82.a2.v1 (http://soybase.org).

### 3.4 Haplotype analysis of candidate genes

**Figure 4:**
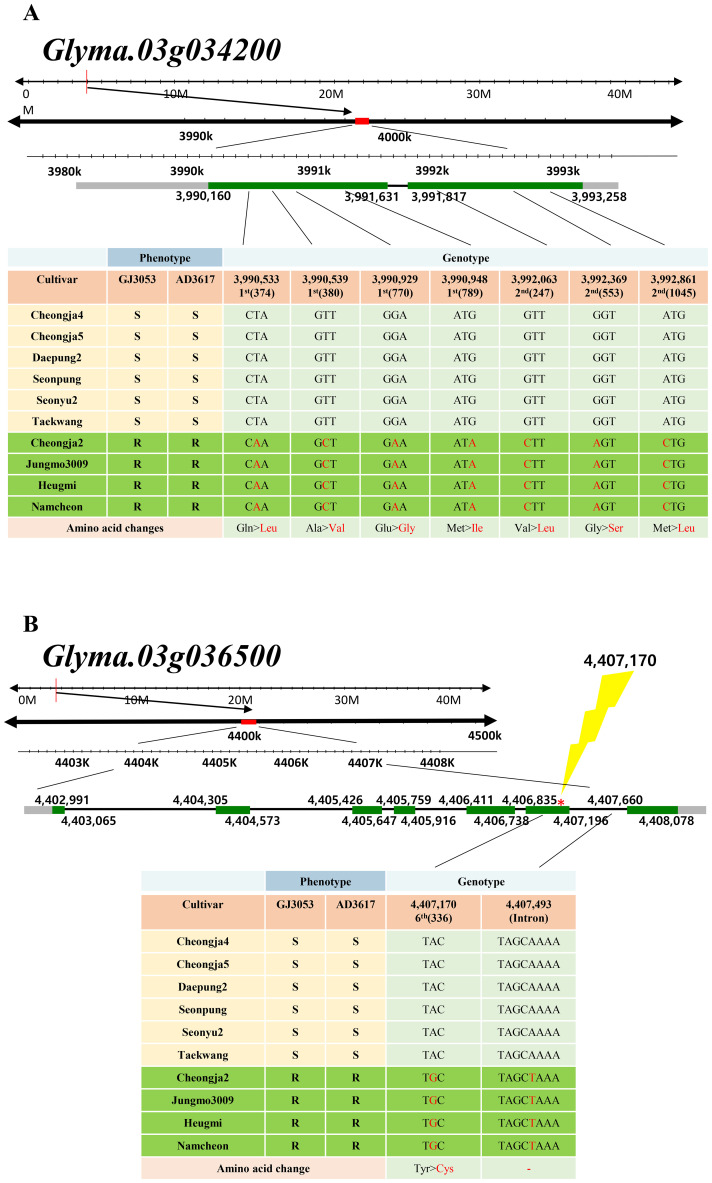
Physical positions of *Glyma.03g034200* **(A)** and *Glyma.03g036500* **(B)** coding sequences in representative resistance and susceptible soybean cultivars infected with *P. sojae* GJ3053 and AD3617. Lightening symbol highlights the position of a key SNP variant identified in *Glyma 03g036500*, which shows a clear association with resistance. Green boxes: exons, black lines: introns, gray boxes: 3’UTR or 5’UTR. SNPs are positioned relative to the genomic position in the Wm82.a2. Gln, glutamine; Ser, serine; Glu, glutamic acid; Pro, proline; Gly, glycine; Ala, alanine; Cys, cysteine; Val, valine; Leu, leucine; Met, methionine; Ile, Isoleucine; Tyr, tyrosine.

The haplotype analysis was performed for fourteen candidate genes on chromosome 3, using four Korean accessions with the highest survival rates as representative resistant accessions (Cheongja2, Jungmo3009, Heugmi, and Namcheon) and six accessions with the lowest survival rates as representative susceptible accessions (Cheongja4, Cheongja5, Daepung2, Seonpung, Seonyu2, and Taekwang). The candidate genes showing significant SNP variants were selected based on their phenotypic differences and re-sequencing genotypes. Of the fourteen candidate genes, only two, *Glyma.03g034200* and *Glyma.03g036500*, showed significant SNP variants. Seven and one non-synonymous SNPs were identified within the exons of *Glyma.03g034200* and *Glyma.03g036500*, respectively. These variants are likely associated with disease resistance, as they correspond to significant survival rate differences between resistant and susceptible groups (**Figure 4**). The lightning symbol highlights the location of the main SNP variation identified in *Glyma 03g036500*, which shows a clear association with resistance (**Figure 4B**).

### 3.5 Expression patterns of resistance genes

**Figure 5:**
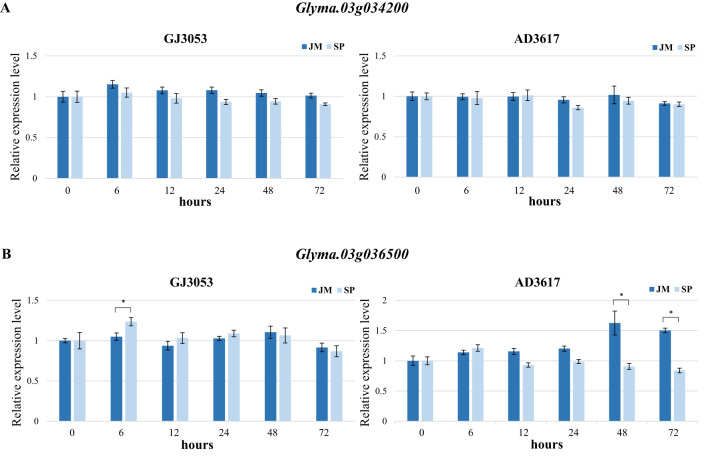
Expression patterns of *Glyma.03g034200* **(A)** and *Glyma.03g036500* **(B)** in Jungmo3009 (JM, Resistant) and Seonpung (SP, Susceptible) during *P. sojae* GJ3053 and AD3617 infection. Samples collected at 0, 6, 12, 24, 48, and 72 hours after inoculation. Three biological replicates were performed, with three technical replicates each. Error bars indicate the standard error of the biological replicates. *p <0.05 (Student’s *t*-test).

To investigate the potential involvement of candidate genes associated with *P. sojae* GJ3053 and AD3617 resistance, the expression patterns of *Glyma.03g034200* and *Glyma.03g036500* were evaluated in Seonpung (S) and Jungmo3009 (R) using qRT-PCR. Differential expression patterns between resistant and susceptible lines may indicate a possible role of these genes in the resistance response. No difference was observed in the expression of *Glyma.03g034200* following inoculation of Seonpung (S) with GJ3053 or AD3617. The expression of *Glyma.03g036500* (GJ3053) was 1.2-fold higher in Seonpung (S) than in Jungmo3009 (R) 6 hours after treatment. The expression of *Glyma.03G036500* (AD3617) in Jungmo3009 (R) was higher than in Seonpung (S) at 48 and 72 hours after treatment, reaching a maximum increase of approximately1.6-fold at 48 hours (**Figure 5**). Hence, *Glyma.03G036500* may be involved in disease defense mechanisms. The levels of *PR* genes activated during infection were monitored for 0**–**72 hours after treatment (Jiang et al., 2015; Zhao et al., 2017). The expression of *Glyma.PRP* increased in Jungmo3009 (R) and Seonpung (S) after 6 h of treatment with GJ3053, and in Seonpung (S) after 72 hours with AD3617. The expression of *Glyma.ERF113* increased more than 10-fold in both samples, starting 24 hours and 48 hours after treatment with GJ3053 and AD3617, respectively. The expression of *Glyma.PRP*, which is involved in *P. sojae* pathogenesis, and *Glyma.ERF113*, which is related to ethylene production during disease response, confirmed that Jungmo3009 (R) and Seonpung (S) were infected with *P. sojae* (**Supplementary Figure 3**).

### 3.6 Alleles associated with resistance

**Figure 6:**
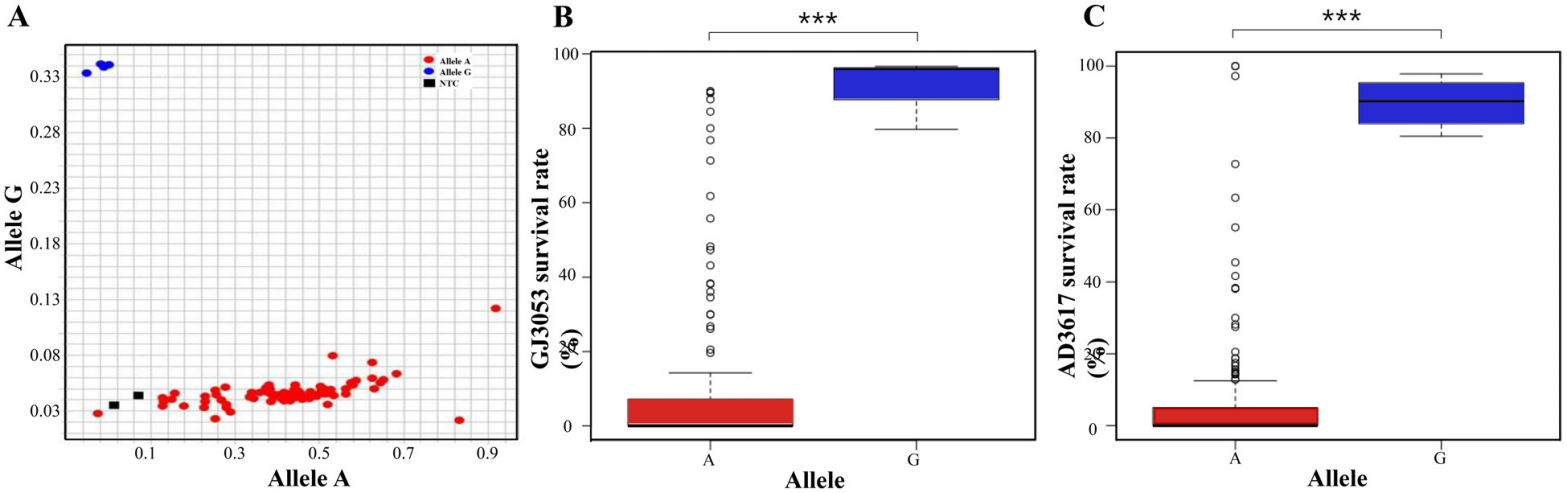
Kompetitive allele specific PCR (KASP) analysis of *Glyma.03g036500* **(A)** Clusters of accessions with the G and **(A)** Blue: allele G, red: allele A, black: no template control (NTC). **(B, C)** Survival rates associated with each allele under GJ3053 **(B)** and AD3617 **(C)** infection. ***p <0.001 (Student’s *t*-test).

We screened 205 soybean accessions using KASP marker of *Glyma.03G036500* (**Supplementary Table 5**). The resistant cultivars Cheongja2, Jungmo3009, Heugmi, and Namcheon all displayed the G allele associated with resistance to both GJ3053 and AD3617. In contrast, most accessions associated with the A allele were susceptible to more than one isolate (**Figures 6B, C**). This highlights the strong association of the G allele with resistance to *P. sojae* and its potential as a marker for screening resistance (**Figure 6**).

## 4 Discussion

### 4.1 Overlapping candidate genes within the *Rps1* allele

For isolates GJ3053 and AD3617, ten and seven accessions exhibited resistance, respectively, with only four (Cheongja2, Heugmi, Jungmo3009, and Namcheon) showing resistance to both GJ3053 and AD3617 (**Figure 1**). GWAS using MLM uncovered a significant overlapping region on chromosome 3 (AX-90410433; 3,628,549 bp), coinciding with previously identified *Rps* regions (*Rps1*, *7*, *Waseshiroge*, *YU25*, *UN1*, *YD29*, *Q*, *DW*, and *SDB*) and candidate genes related to these regions (Demirbas et al., 2001; Weng et al., 2001; Sugimoto et al., 2011; Sun et al., 2011; Lin et al., 2013; Zhang et al., 2013; Li et al., 2017; Niu et al., 2017; Zhong et al., 2018; Jang et al., 2020a; You et al., 2023b). For GJ3053 and AD3617, an overlapping region was identified on chromosome 3 between 2.9 and 4.4 Mbp, encompassing six disease-related genes: *Glyma.03g027200* [LRR receptor-like protein kinase (RLK) family protein], *Glyma.03g032300* (LRR-RLK), *Glyma.03g033900* (regulation of cyclin-dependent STK), *Glyma.03g034200* (LRR protein kinase family protein), *Glyma.03g036000* (STK), and *Glyma.03g036500* (STK) (**Table 2**).*Glyma.03g027200* is a potential gene in *RpsQ* (Li et al., 2017; Zhong et al., 2019). Schneider et al. (2016) also identified *Glyma.03g034200* (LRR) among six candidate genes in the GWAS Gapit analysis of 797 germplasms with partial resistance to *P. sojae*, likely through weak *R*-gene-like responses or other defense-related pathways (Schneider et al., 2016). Haplotypes analysis of *Glyma.03g034200* identified four amino acid variants in the first and three in the second exon consistent with the phenotypes of the resistant and susceptible cultivars (**Figure 4**). *Glyma.03g034200* was found to overlap with resistance-associated genomic regions in all four isolates collected from South Korea. The QTL *RpsDW* (3.8–4.6 Mbp) confers resistance to isolate 2457 in the Daepung/Daewon RIL population (Jang et al., 2020a). In isolate 2858, the QTL region in the Daepung/Saedanbaek RIL population overlapped with *RpsSDB* (3.3–4.3 Mbp) (You et al., 2023b). The SNP at 3,990,383 bp observed in the Daepung/Daewon RIL population was not detected in our study (Jang and Lee, 2021). In several studies using the hypocotyl inoculation method and the layer test to evaluate race-specific and partial resistance, *Glyma.03g034200* was found to be associated with *P. sojae* (Schneider et al., 2016; Jang et al., 2020a; You et al., 2023b). However, qRT-PCR in this study did not reveal any changes in its expression from 0 to 72 hours after treatment (**Figure 5A**). *R-gene* expression levels can vary, and some may exhibit weak expression even while still contributing to resistance. Partial resistance, in particular, often involves complex regulatory networks with subtle gene expression changes (Jang and Lee, 2020; Chandra et al., 2022). However, gene expression analysis alone is not sufficient to determine its specific resistance mechanism. To further clarify its role, additional experiments are needed to elucidate its precise mechanism in soybean defense*. Glyma.03g036000* is also a candidate gene within *RpsGZ* in the Guizao1/BRSMG68 RIL population (Jiang et al., 2020). Among the candidate genes, *Glyma.03g036500* expression correlated with the *P. sojae* resistance phenotype. A tyrosine-cysteine substitution SNP (4,407,170 bp) was identified in the sixth exon, particularly in four resistant accessions, Cheongja2, Jungmo3009, Heugmi, and Namcheon (**Figure 4**). KASP marker analysis for this SNP indicated that Cheongja2, Heugmi, Jungmo3009, and Namcheon carried the G allele associated with resistance (**Figure 6**). Further, accessions that were sensitive to more than one isolate carried the A allele. This is the first study to identify G*lyma.03g036500* as a candidate gene for *P. sojae* resistance. G*lyma.03g036500* was predicted to encode an STK, which participates in plant resistance, and STK-LRRs facilitate transmembrane signal transduction (Hardie, 1999; Martin et al., 2003). Previous studies identified two Toll Interleukin Receptor-nucleotide-binding–LRR resistance proteins in the defense response against soybean mosaic virus (Karthikeyan et al., 2018). Recent research has shown that these RGAs, predicted to encode STKs, may be associated with *Rps* alleles/genes (Niu et al., 2017; Zhong et al., 2018; Jang et al., 2020a; Jiang et al., 2020; Karhoff et al., 2022; You et al., 2023b). These alleles, found in gene-dense regions, are located near genes encoding NBS-LRR proteins and STKs, which are considered RGAs. In other crops, such as rice and wheat, many *R*-genes (e.g., *Xa21, Xa21D, Xa26*, and *Lr10*) share structures similarities with STKs (Song et al., 1995; Feuillet et al., 1997; Wang et al., 1998; Sun et al., 2004).

Our findings indicated that the resistance region for GJ3053 and AD3617 was located at 2.9– 4.4 Mbp. The identified soybean resistance genes for *P. sojae* overlap with previously reported *Rps* regions on chromosome 3, including *Rps 1, 7, Waseshiroge, YU25, UN1, YD29, DW, and SDB*. *Glyma.03g036500* was located within *Rps 1, 7, YU25*, and *DW* and may play a significant role in resistance against GJ3053 and AD3617 (Demirbas et al., 2001; Weng et al., 2001; Sun et al., 2011; Sugimoto et al., 2011; Jang et al., 2020a; You et al., 2023b). Therefore, *Glyma.03g036500* may serve as a valuable marker for selecting resistance resources in the breeding of elite soybean cultivars.

### 4.2 Resistance genes for PRSR located on chromosomes 13, 14, and 16

GWAS identified SNPs on chromosomes 13, 14, and 16, in addition to chromosome 3. AX-90525316 (30,033,688bp) on chromosome 13 was related to AD3617 resistance; RGAs within the LD region included *Glyma.13g181400*, *Glyma.13g184200, Glyma.13g187600*, *Glyma.13g188800, Glyma.13g188900*, and *Glyma.13g189000* (**Tables 1**, **2**). Near this locus, *Glyma.13g184800* was reported by Qin et al. (2017), and *Glyma.13g190400* was identified in the PI449459/Misty RIL population (Zhong et al., 2020). These findings further support the association between these candidate genes and *P. sojae* resistance. AX-90327146, AX-90397007, AX-90391625, and AX-90399355 were detected on chromosome 14 and appeared to confer resistance specifically to GJ3053. LD analysis of these significant SNP regions revealed distances of 17.5–19.9, 35.3–36.3, and 39.9–40.9 Mbp on chromosome 14 (**Table 1**). Five candidate genes related to *P. sojae* resistance were identified in these regions: *Glyma.14g124400* and *Glyma.14g124700*, related to LRR-RLKs, and *Glyma.14g164400*, *Glyma.14g165700*, and *Glyma.14g166000*, related to STKs (**Table 2**). A few genes on chromosome 14 have been previously associated with *P. sojae* resistance. A previous GWAS of 189 soybean germplasms indicated that *Glyma.14g079500* and *Glyma.14g079600*, which encode proteins homologous to the broad-spectrum mildew resistance protein RPW8 in Arabidopsis, were associated with *P. sojae* resistance (Qin et al., 2017). Additionally, Van et al. (2021) reported SNP associated with *P. sojae* resistance at position 47,590,507 bp on chromosome 14. These findings, along with our results, highlight the potential role of chromosome 14 in *P. sojae* resistance mechanisms. On chromosome 16, AX-90331170 (3,163,817 bp) and AX90449650 (8,750,924 bp) were associated with GJ3053 and AD3617, respectively; however, these regions did not overlap (**Table 1**). The final candidate genes included *Glyma.16g032700* and *Glyma.16g034900* in GJ3053 and *Glyma.16g078800, Glyma.16g078900*, *Glyma.16g079000, Glyma.16g079200*, and *Glyma.16g081700* in AD3617 (**Table 2**). A previous GWAS of 224 germplasm accessions identified *Glyma.16g30140, Glyma.16g04700, Glyma.16g14080*, and *Glyma.16g31930* (Glyma.v1) (Huang et al., 2016). These findings suggest that as new pathotypes arise, resistance genes on several chromosomes diversify. The variation in *Rps* gene expression highlights the complexity of genetic interactions in *P. sojae* resistance.

In South Korea, the cultivation area of soybeans in paddy fields expanded from 4,422 ha in 2016 to 22,438 ha in 2024 (Statistics Korea, 2024). High-moisture soils, such as those in paddy fields, create a favorable environment for PRSR infection because of water saturation, thus significantly increasing the risk of disease occurrence. Therefore, establishing robust management strategies focused on developing PRSR-resistant cultivars is essential.

Our study employed a GWAS to identify candidate genes associated with *P. sojae* resistance and highlighted the applicability of KASP markers for selecting resistant accessions. The use of genetic markers facilitates the rapid identification of resistance resources in early-generation lines, thereby accelerating the development of resistant cultivars. Furthermore, future studies should consider using transformation or gene-editing techniques to validate the identified candidate genes. This study represents a crucial advance in the characterization of soybean resistance to *P. sojae* and provides novel insights into the associated genetic mechanisms, such as the involvement of *Glyma.03g036500*.

## Data Availability

The data presented in the study are deposited in the National Agricultural Biotechnology Information Center (NABIC) for Database repository (https://nabic.rda.go.kr/), accession number NN-8916, NN-8918, NN-8924, and NN-8946.
