# Supplementary material for: Identification and genetic analysis of candidate genes for resistance against Phytophthora sojae in soybean using a genome-wide association study
Source: Front Plant Sci. 2025 May 2;16:1520999. doi: 10.3389/fpls.2025.1520999 (PMC12081341; doi:10.3389/fpls.2025.1520999)
Supplement: Supplementary file 1 [file SupplementaryFile1.docx]

Identification and Genetic Analysis of Candidate Genes for Resistance against *Phytophthora sojae* in Soybean using a Genome-wide Association Study

**Hye Rang Park^1^, Su Vin Heo^1*^, Beom Kyu Kang^1^, Hyoseob Seo^1^, Eunsoo Lee^1^, Jihee Park^1^, Yun Woo Jang^2^, Jeong Hyun Seo^1^, Girim Park^1^, Jun Hoi Kim^1^, Yeong Hoon Lee^1^, Won Young Han^1^, Myung Chul Seo^1^, and Ji-Ung Jeung^1^**

^1^Division of Upland Crop Breeding Research, Department of Southern Area Crop Science, National Institute of Crop Science, Rural Development Administration, Miryang 50424, Republic of Korea

^2^Division of Crop Production Technology Research, Department of Southern Area Crop Science, National Institute of Crop Science, Rural Development Administration, Miryang 50424, Republic of Korea

Supplementary Material

# Supplementary Figures and Tables

## Supplementary Figures


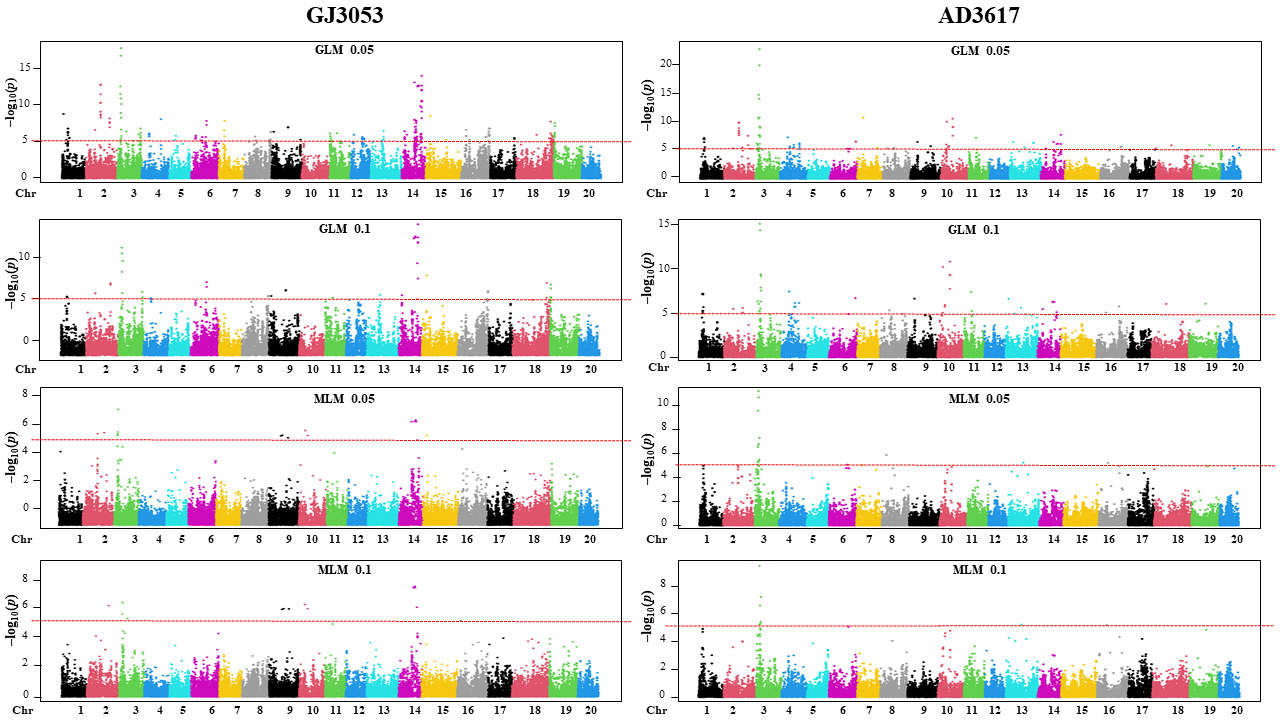


**Supplementary Figure 1.** Manhattan plots of GWAS for *P. sojae* GJ3053 and AD3617 resistance. Red dashed lines: significance threshold (–log_10_(*p*) = 5). GLM 0.05: general linear model, minor allele frequency < 5%; GLM 0.1: general linear model minor, allele frequency < 10%; MLM 0.05: mixed linear model, minor allele frequency < 5%; MLM 0.1: mixed linear model, minor allele frequency < 10%.


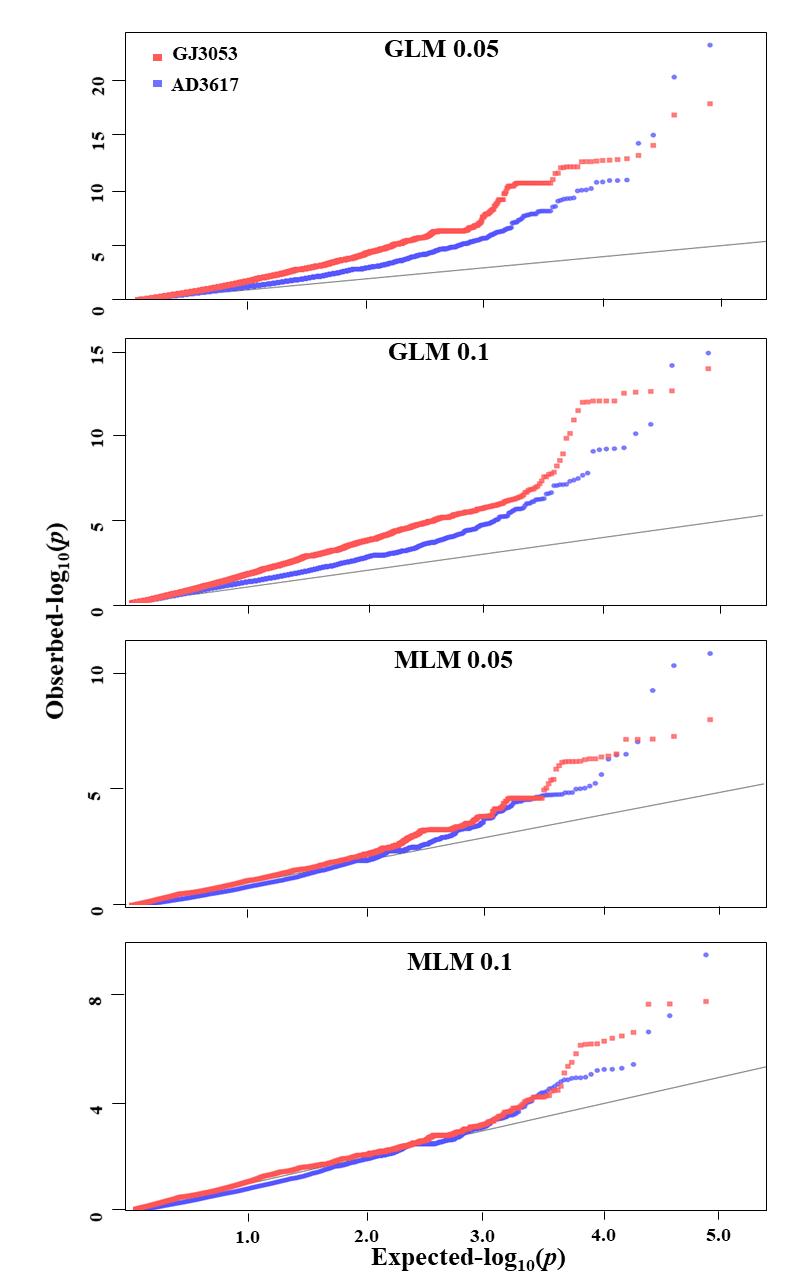


**Supplementary Figure 2.** Quantile-quantile (QQ) plots of GWAS for *P. sojae* GJ3053 and AD3617 resistance. GLM 0.05: general linear model, minor allele frequency < 5%; GLM 0.1: general linear model, minor allele frequency < 10%; MLM 0.05: mixed linear model, minor allele frequency < 5%; MLM 0.1: mixed linear model, minor allele frequency < 10%.

**
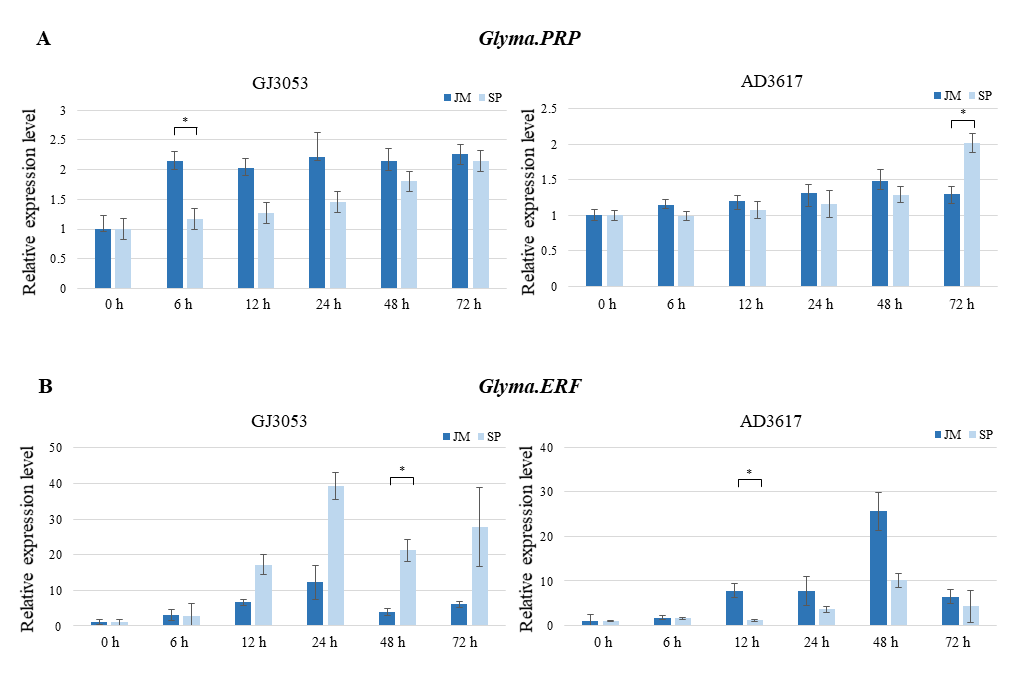
Supplementary Figure 3.** Expression patterns of *Glyma.PRP* and *Glyma.ERF* in Jungmo3009 (JM, resistant) and Seonpung (SP, susceptible) during *P. sojae* GJ3053 and AD3617 infection. Infected samples were collected 0, 6, 12, 24, 48, and 72 hours after inoculation. Three biological replicates were performed, with three technical replicates each. Error bars indicate the standard error of biological replicates. **p* <0.05 (Student’s *t*-test).

## Supplementary Tables

**Supplementary Table 1**. List of 205 soybean accessions and survival rates following inoculation with *P. sojae* GJ3053 and AD3617

**Supplementary Table 2.** Summarized genotype data from GWAS of 205 soybean accessions.

|  | GLM 0.05^1^ | GLM 0.1^2^ | MLM 0.05^3^ | MLM 0.1^4^ |
| --- | --- | --- | --- | --- |
| Minor Allele Frequency (%) | 5 | 10 | 5 | 10 |
| Number of Taxa | 205 | 205 | 205 | 205 |
| Number of Sites | 79101 | 65127 | 79101 | 65127 |
| Sites x Taxa | 1.62E+07 | 1.34E+07 | 1.62E+07 | 1.34E+07 |
| Number Not Missing | 1.62E+07 | 1.33E+07 | 1.62E+07 | 1.33E+07 |
| Proportion Not Missing | 0.99613566 | 0.996017313 | 0.99613566 | 0.996017313 |
| Number Missing | 62663 | 53173 | 62663 | 53173 |
| Proportion Missing | 0.00386434 | 0.003982687 | 0.00386434 | 0.003982687 |
| Number Gametes | 3.24E+07 | 2.67E+07 | 3.24E+07 | 2.67E+07 |
| Gametes Not Missing | 3.23E+07 | 2.66E+07 | 3.23E+07 | 2.66E+07 |
| Proportion Gametes Not Missing | 0.99613566 | 0.996017313 | 0.99613566 | 0.996017313 |
| Gametes Missing | 125326 | 106346 | 125326 | 106346 |
| Proportion Gametes Missing | 0.00386434 | 0.003982687 | 0.00386434 | 0.003982687 |
| Number Heterozygous | 164744 | 142380 | 164744 | 142380 |
| Proportion Heterozygous | 0.010159534 | 0.010664342 | 0.010159534 | 0.010664342 |
| Average Minor Allele Frequency | 0.241533854 | 0.277429077 | 0.241533854 | 0.277429077 |
| No. of SNPs used in GWAS Analysis | 79,101 | 65,127 | 79,102 | 65,128 |
| log_10_(1/SNP) | 4.898182 | 4.813761 | 4.898182 | 4.813761 |

^1^GLM 0.05: general linear model, minor allele frequency < 5%; ^2^GLM 0.1: general linear model, minor allele frequency < 10%; ^3^MLM 0.05: mixed linear model, minor allele frequency < 5%; ^4^MLM 0.1: mixed linear model, minor allele frequency < 10%.

**Supplementary Table 3.** Representative resistance and susceptible soybean cultivars against *P. sojae* GJ3053 and AD3617

| Genotype | GJ3053 |  | AD3617 |
| --- | --- | --- | --- |
| Cheongja2 | R^1^ |  | R |
| Heugmi | R |  | R |
| Jungmo3009 | R |  | R |
| Namcheon | R |  | R |
| Cheongja4 | S^2^ |  | S |
| Cheongja5 | S |  | S |
| Daepung2 | S |  | S |
| Seonpung | S |  | S |
| Seonyu2 | S |  | S |
| Taekwang | S |  | S |

^1^R: resistant, survival rate > 80%; ^2^S: susceptible, survival rate < 20%.

**Supplementary Table 4**. Primer list for qRT-PCR

| Gene ID | Forward primer | Reverse primer |
| --- | --- | --- |
| *Glyma.03g034200* | GTTCAGAACTAACATTTCTGAATCTTTCTCACAACAAGC | CACCTAGGAGGTGGTTGTACG |
| *Glyma.03g036500* | GCTCAAGATTGGCCTCAAGATGAACTATTCATGAATG | CTGCTCCTAGTGCTAGAGTTGGATCATGCTC |
| *GmPRP* | TTCAGCCTAAACGGAAGGAAGCCT | TTGTCGTGAAGGCCTTATGGGATG |
| *GmERF113* | GATAGCACCCTTTCTTCACCAA | ATGTCTTTTTCTCCCATTTCCT |
| *Actin11* | ATCTTGACTGAGCGTGGTTATTCC | GCTGGTCCTGGCTGTCTCC |

**Supplementary Table 5.** Primer for KASP analysis.

| *Glyma. 03g036500* | | | CG% | | | |
| --- | --- | --- | --- | --- | --- | --- |
| Susceptibility  Allele A | Resistance  Allele G | Primer Common | X | Y | Common | |
| GTTCCCACCATCGTGAGAATCATTA | TTCCCACCATCGTGAGAATCATTG | GTACCTTGAGGCCAATCTTGAGCAA | 44.0 | 45.8 | | 48.0 |

**Supplementary Table 6.** Phenotypic analysis of survival rates associated with GJ3053 and AD3617 infection.

| Isolates | Mean | Min% | Max | Stdev | Var | Kur | Skew |
| --- | --- | --- | --- | --- | --- | --- | --- |
| GJ3053 | 9.7 | 0 | 96.7 | 21.8 | 475.3 | 7.2 | 2.8 |
| AD3617 | 7.6 | 0 | 100 | 19.3 | 372.6 | 12.7 | 3.6 |

^1^Min : Minimum, ^2^Max : Maximum, ^3^Stdev : standard deviation, ^4^Var : Variance, ^5^Kur : Kurtosis, ^6^Skew : Skewness.

**Supplementary Table 7.** Significant SNPs associated with resistance to *P. sojae* GJ3053 and AD3617 based on GWAS

| **Isolate** | **Chr**^1^ | **SNP ID** | **Physical**  **Position**^2^  **(bp)** | **GLM_0.05**^3^ | | | **GLM_0.1**^4^ | | | **MLM_0.05**^5^ | | | **MLM_0.1**^6^ | | |
| --- | --- | --- | --- | --- | --- | --- | --- | --- | --- | --- | --- | --- | --- | --- | --- |
|  |  |  |  | ***p*** | **LOD** | **R^2^** | ***p*** | **LOD** | **R^2^** | ***p*** | **LOD** | **R^2^** | ***p*** | **LOD** | **R^2^** |
|  | 2 | AX-90438121 | 34,224,450 |  |  |  |  |  |  | 3.12E-07 | 6.505845406 | 0.1594 | 4.32E-07 | 6.364184 | 0.15565 |
| GJ3053 | 3 | AX-90354028 | 3,417,978 | 3.19E-12 | 11.49655 | 0.2336 | 3.19E-12 | 11.49655 | 0.2336 | 2.89E-06 | 5.539177 | 0.13538 | 1.61E-06 | 5.793336 | 0.14181 |
|  |  | AX-90410433 | 3,628,549 | 1.16E-11 | 10.93569 | 0.22066 | 1.16E-11 | 10.93569 | 0.22066 | 2.83E-07 | 6.547937 | 0.15942 | 2.66E-07 | 6.575739 | 0.16015 |
|  |  | AX-90391625 | 35,814,319 | 2.98E-13 | 12.52619 | 0.24948 | 2.98E-13 | 12.52619 | 0.24948 | 5.21E-08 | 7.283479 | 0.17893 | 2.38E-08 | 7.622876 | 0.18803 |
|  | 9 | AX-90395336 | 20,563,970 |  |  |  |  |  |  | 5.34E-07 | 6.272597 | 0.15222 | 7.80E-07 | 6.108134 | 0.14795 |
|  |  | AX-90347843 | 31,690,000 |  |  |  |  |  |  | 4.73E-07 | 6.325359 | 0.15699 | 7.03E-07 | 6.153298 | 0.15236 |
|  |  | AX-90482872 | 40,718,457 |  |  |  |  |  |  | 7.29E-07 | 6.137433 | 0.15191 | 7.28E-07 | 6.13769 | 0.15142 |
|  | 10 | AX-90467453 | 9,112,938 |  |  |  |  |  |  | 2.28E-07 | 6.642427 | 0.16301 | 3.58E-07 | 6.446226 | 0.15785 |
|  |  | AX-90331570 | 13,685,110 |  |  |  |  |  |  | 4.86E-07 | 6.313632 | 0.15355 | 6.92E-07 | 6.160126 | 0.14953 |
|  | 14 | AX-90327146 | 18,058,454 | 2.11E-13 | 12.67609 | 0.25098 | 2.11E-13 | 12.67609 | 0.25098 | 4.01E-08 | 7.396401 | 0.18187 | 1.92E-08 | 7.715998 | 0.19044 |
|  |  | AX-90397007 | 19,400,109 | 2.56E-13 | 12.59191 | 0.24954 | 2.56E-13 | 12.59191 | 0.24954 | 5.35E-08 | 7.271784 | 0.17855 | 2.43E-08 | 7.614483 | 0.18771 |
|  |  | AX-90399355 | 40,454,708 | 1.43E-10 | 9.844755 | 0.20104 | 1.43E-10 | 9.844755 | 0.20104 | 1.04E-06 | 5.983259 | 0.14471 | 5.53E-07 | 6.257126 | 0.15182 |
|  | 16 | AX-90331170 | 3,163,817 |  |  |  |  |  |  |  |  |  | 4.47E-06 | 5.349692 | 0.13906 |
| AD3617 | 3 | AX-90432113 | 3,285,331 | 1.19E-15 | 14.9229 | 0.27125 | 1.19E-15 | 14.9229 | 0.27125 | 4.11E-10 | 9.385990 | 0.21169 | 3.73E-10 | 9.427800 | 0.21281 |
|  |  | AX-90410433 | 3,628,549 | 6.55E-15 | 14.18383 | 0.27629 | 6.55E-15 | 14.18383 | 0.27629 | 2.54E-07 | 6.595423 | 0.16066 | 2.54E-07 | 6.594910 | 0.16065 |
|  |  | AX-90402933 | 5,210,627 | 3.49E-08 | 7.456652 | 0.13944 | 3.49E-08 | 7.456652 | 0.13944 | 4.27E-06 | 5.369542 | 0.10949 | 3.96E-06 | 5.401833 | 0.11027 |
|  |  | AX-90365087 | 5,287,030 | 8.48E-10 | 9.071717 | 0.17041 | 8.48E-10 | 9.071717 | 0.17041 | 6.88E-08 | 7.162475 | 0.15395 | 6.42E-08 | 7.192255 | 0.15472 |
|  | 13 | AX-90525316 | 30,033,688 | 2.90E-06 | 5.537063 | 0.11859 | 2.90E-06 | 5.537063 | 0.11859 | 7.31E-06 | 5.135940 | 0.12300 | 6.04E-06 | 5.218740 | 0.12510 |
|  | 16 | AX-90449650 | 8,750,924 | 9.95E-06 | 5.002077 | 0.10828 | 9.95E-06 | 5.002077 | 0.10828 | 7.58E-06 | 5.120422 | 0.12276 | 6.63E-06 | 5.178205 | 0.12423 |

^1^Chr: chromosome, ^2^The physical positions are based on soybean genome W82.a2.v1 (http://soybase.org), ^3^GLM 0.05: general linear model, minor allele frequency < 5%; ^4^GLM 0.1: general linear model, minor allele frequency < 10%; ^5^MLM 0.05: mixed linear model, minor allele frequency < 5%; ^6^MLM 0.1: mixed linear model, minor allele frequency < 10%.
